# Impact on Patient Outcomes of Continuous Vital Sign Monitoring on Medical Wards: Propensity-Matched Analysis

**DOI:** 10.2196/66347

**Published:** 2025-03-11

**Authors:** Bradley Rowland, Amit Saha, Vida Motamedi, Richa Bundy, Scott Winsor, Daniel McNavish, William Lippert, Ashish K Khanna

**Affiliations:** 1 Department of Internal Medicine Section of Hospital Medicine Wake Forest University School of Medicine Winston-Salem, NC United States; 2 Department of Anesthesiology Wake Forest University School of Medicine Winston Salem, NC United States; 3 Department of Anesthesiology Perioperative Outcomes and Informatics Collaborative Winston-Salem, NC United States; 4 Department of Anesthesiology Outcomes Research Consortium Cleveland, OH United States; 5 Department of Anesthesiology Vanderbilt University Medical Center Nashville, TN United States; 6 Corewell Health Frederik Meijer Heart & Vascular Institute Michigan State University Grand Rapids, MI United States

**Keywords:** clinical, continuous, monitoring, outcomes, medical ward, wireless, wireless monitoring, vital sign, ward, patient outcome, hospital ward, clinical outcome, contemporaneous control, contemporaneous, teenager, young adult, adult, monitoring device, wireless device, wearable, patient monitoring

## Abstract

**Background:**

Continuous and wireless vital sign (VS) monitoring on hospital wards is superior to intermittent VS monitoring at detecting VS abnormalities; however, the impact on clinical outcomes remains to be confirmed. A recent propensity-matched study of primary surgical patients found decreased odds of intensive care unit (ICU) admission and mortality in patients receiving continuous monitoring. Primary surgical patients are inherently different from their medical counterparts who typically have high morbidity, including frailty. Continuous monitoring research has been limited in primary medical patients.

**Objective:**

This study aims to evaluate the clinical outcomes of primary medical patients who received either continuous or, as a contemporaneous control, intermittent vital monitoring as the standard of care using propensity matching.

**Methods:**

Propensity-matched analysis of a population-based sample of 7971 patients admitted to the medical wards between January 2018 and December 2019 at a single, tertiary United States medical center. The continuous monitoring device measures oxygen saturation, heart rate, respiratory rate, continuous noninvasive blood pressure, and either 3-lead or 5-lead electrocardiogram. Patients received either 12 hours or more of continuous and wireless VS monitoring (n=1450) or intermittent VS monitoring (n=6521). The primary outcome was the odds of a composite of in-hospital mortality or ICU transfer during hospitalization. Secondary outcomes were the odds of individual components of the primary outcome, as well as heart failure (HF), myocardial infarction (MI), acute kidney injury (AKI), and rapid response team (RRT) activations.

**Results:**

Those who received intermittent VS monitoring had greater odds of a composite of in-hospital mortality or ICU admission (odds ratio [OR] 2.79, 95% CI 1.89-4.25; *P*<.001) compared with those who had continuous and wireless VS monitoring. The odds of HF (OR 1.03, 95% CI 0.83-1.28; *P*=.77), MI (OR 1.58, 95% CI 0.77-3.47; *P*=.23), AKI (OR 0.74, 95% CI 0.62-1.02; *P*=.06), and RRT activation (OR 0.94, 95% CI 0.75-1.19; *P*=.62) were similar in both groups.

**Conclusions:**

In this propensity-matched study, medical ward patients who received standard of care intermittent VS monitoring were at nearly 3 times greater odds of transfer to the ICU or death compared with those who received continuous VS monitoring. Our study was primarily limited by the inability to match patients on admission diagnosis due to limitations in electronic health record data. Other limitations included the number of and reasons for false alarms, which can be challenging with continuous monitoring strategies. Given the limitations of this work, these observations need to be confirmed with prospective interventional trials.

## Introduction

Vital sign (VS) monitoring is routine practice on the general medical wards and is typically performed as an intermittent “spot check,” at 4 to 8-hour intervals [1]. However, this intermittent VS monitoring may fail to detect VS abnormalities [2-5], and perturbations in VS may be sustained for long periods between spot check intervals [6,7]. The failure to detect abnormal VS or their trends has significant implications for clinical care [8]. For example, transient hypotension is associated with worse outcomes [9-14]. Furthermore, the failure to act on or recognize deterioration was identified as the most common reason for harm in a study of 2010 hospitalized patient deaths in the United Kingdom between 2010 and 2012 [15].

Aggregate VS scoring systems such as the early warning score (EWS) have been developed to aid in the identification and risk stratification of patients at risk of or experiencing clinical deterioration. Once identified, clinical response teams, often termed “rapid response teams” (RRTs), may intervene to provide stabilization or transfer to a higher level of care. This identification and response have been described as the afferent and efferent arms, respectively, of rapid response [16]. The afferent response is contingent upon appropriate and timely recognition of clinical deterioration, which is accomplished primarily through the detection of abnormal VS. The use of intermittent VS monitoring may fail to detect clinical deterioration, thereby precluding or delaying this process.

The advent of wearable and wireless devices capable of continuous VS monitoring integrated into the clinical workflow offers a solution to the failure-to-detect paradigm associated with intermittent VS monitoring; however, clinical outcome data are needed. Evidence regarding outcomes with continuous VS monitoring relies almost exclusively on before-and-after comparison studies, and the research has also focused primarily on surgical patients [17]. Two systematic reviews of continuous VS monitoring concluded that existing study quality is low-to-moderate and does not show benefit of continuous compared with intermittent VS monitoring [18,19]. However, these reviews were limited by the heterogeneity of devices included in the studies and narrow scope of the patient populations. That is, the analyses included over 15 different device types making cross comparison and generalization challenging. Additionally, the analyses included almost entirely surgical populations, with individual studies including niche surgical areas such as gastrointestinal cancer, again limiting the generalizability of the analyses.

The granular detection capabilities of continuous and wireless VS monitoring mitigate the variability that is inevitable in today’s complex health care systems, allowing capture and intervention of clinical deterioration to improve quality of care.

At Atrium Health Wake Forest Baptist Medical Center, continuous VS monitoring using wearable and portable monitoring device technology on surgical and medical wards has been in place since 2015 and is coupled with VS threshold–based alarms and interventions based on best practices. An earlier report from our institution demonstrated a significant decrease in RRT calls after implementation [20,21]. We have also reported substantial changes in heart rate (HR) and blood pressure (BP) detected by these monitoring devices that would have gone undetected with traditional intermittent VS monitoring. Furthermore, we recently reported a propensity-matched analysis of 36,000 surgical ward patients, where 12,345 intermittently monitored patients were compared with 7955 continuously monitored patients, and a nearly 2 times increased odds of intensive care unit (ICU) admission or in-hospital mortality was seen in the intermittently monitored group [22]. The inherent differences between a primary surgical population, where nearly all continuous postprocedure monitoring research has been conducted, and a primary medical population is paramount to the significance of this work. Primary medical admissions are inherently different from surgical ones. Medical patients often have multiple acute problems related to single or multiple intersected disease processes. Often, patients admitted to a medical service are deemed too sick or high risk to undergo surgery.

Therefore, we sought to evaluate and compare clinical outcomes in a large propensity-matched medical ward population admitted to a single institution who received either continuous or, as a contemporaneous control, intermittent VS monitoring as the standard of care every 4 hours to 8 hours. We hypothesized that earlier detection of VS aberrations through continuous VS monitoring would reduce ICU transfer and in-hospital mortality. Secondary outcomes included heart failure (HF), myocardial infarction (MI), acute kidney injury (AKI), and RRT activations.

## Methods

### Population and Setting

We first identified adult patients (aged ≥18 years) admitted to a tertiary care center (Atrium Health Wake Forest Baptist Medical Center main campus) over 2 years (between January 1, 2018, and December 31, 2019) under a primary medical service.

Primary medical services included hospitalist, general internal medicine, and family medicine. Provider staffing varied by service but included physician attendings, advanced practice providers, and medical residents. Primary medical services represented the typical breadth of patients admitted to hospital medicine services. Admission diagnoses were not included in propensity matching. Patients having any surgery during admission were excluded from analysis. Race and ethnicity were self-described by patients on admission then retrospectively collected with other demographics from the electronic health record (EHR).

### Ethical Considerations

Regarding human subject research ethics review, our study was a secondary analysis and did not actively involve nor enroll human subjects. Our study and a priori defined statistical analysis plan were approved by the Wake Forest University Health Sciences Institutional Review Board (IRB; approval number IRB00083520). Informed consent from human subjects was not obtained for this secondary analysis. Primary consent for data collection was allowed by the IRB without additional consent. Privacy and confidentiality protection were maintained using de-identified data for analysis and using only data needed for the analysis. Data were stored in a secure location per IRB requirements.

### Monitoring Protocols

Patients admitted to the medical wards either received (1) VS monitoring with a continuous monitoring device, ViSi Mobile, (Sotera Wireless Inc), or (2) standard intermittent VS monitoring (every 4-8 hours). Briefly, the ViSi Mobile system is a portable wrist-mounted system that received clearance from the Food and Drug Administration in 2012. These ViSi devices were purchased and implemented by the Wake Forest Baptist Hospital system in 2015. Today, ViSi monitors are on several different medical and surgical units; however, these devices are not available on all units. If units do not have ViSi monitors or patients decline to wear the monitor, then intermittent monitoring is used.

Hospital units with ViSi devices are staffed by the same number of personnel as those not doing this monitoring. Nurse-to-patient ratios on our floors range from 1:3 to 1:6. For this analysis, patients were not randomized into continuous or intermittent VS monitoring. The type of monitoring was dependent upon the floor a patient was admitted to, which was dependent on bed availability and not patient clinical status. Based on clinical experience, there would not have been a significant crossover between groups; that is, once a patient arrived on the floor and was allocated to either continuous or intermittent VS monitoring, they would then most likely continue that strategy until discharge—unless, for example, a patient was transferred to a different unit and received a different type of monitoring strategy there.

The ViSi device records oxygen saturation (SpO_2_), HR, respiratory rate (RR), continuous noninvasive BP, and either 3-lead or 5-lead electrocardiogram. Continuous BP measurements are estimated from pulse arrival time, specifically the time that elapses between the R wave being detected and the arrival of the resulting pulse at the SpO_2_ finger sensor. According to the manufacturer, the estimated maximal mean error for BP measurements is 5 mm Hg with a standard deviation ≤8 mm Hg [23].  

ViSi monitors were calibrated daily with the oscillometric brachial cuff method and connected to the hospital’s wireless network, and measurements were distributed to the central nurse’s station for that unit and continuously displayed. The ViSi monitoring system measures VS nearly continuously, and these data are transmitted to the central nurse’s station for each respective unit; however, these data are not transmitted nor recorded directly in the EHR. That is, nurses or nursing assistants must still see the patient and collect VS from the device or the monitoring station remotely and then manually document (chart) these vitals in the EHR. No matter what, the VS assessment includes direct patient visualization to measure and record patient alertness. The VS data not recorded in the EHR are temporarily stored on the ViSi manufacturer’s cloud for a set period of time and are then destroyed. Unfortunately, we were *only* able to acquire and evaluate the EHR data.

VS abnormalities exceeding established thresholds for the local hospital system generated alerts at the floor’s central nursing station and to the nurses’ hospital-supplied phones (ASCOM). Alarms not addressed by the primary nurses were escalated to other floor nurses, then to the unit manager. Time to escalation depended on the VS and the relative degree of abnormality. For example, SpO_2_ abnormality alerts, if not acknowledged, were quickly escalated over minutes.

EWS at our institution are based on the patient’s weighted VS of HR, RR, SpO_2_, temperature, BP, rate and percentage of inspired oxygen, and level of awareness determined by the patient’s nurse [24]. EWS are calculated by the EHR every 4 hours based on data input from ViSi monitors, which can be collected from the central display or at the bedside, or those collected by intermittent monitoring. Patients, regardless of monitoring strategy, must be observed at bedside to determine level of awareness for EWS calculation. Total scores range from 0 to 20. Ad hoc EWS may be generated by the clinical team in response to changes in a patient’s clinical status through either monitoring strategy. For example, if abnormal VS are detected by ViSi monitoring and an alarm is generated at the central nurse’s station, the clinical team may assess the patient and generate an updated EWS. An RRT activation is triggered if the EWS is ≥8.

### Outcomes

Our primary outcome was the risk of a composite of in-hospital mortality or ICU admission during the hospital encounter. Secondary outcomes were the individual components of the primary outcome (in-hospital mortality and ICU admission), as well as HF, MI, AKI, and RRT activations throughout the hospitalization. Exploratory outcomes included hospital length of stay (LOS), ICU LOS, and median EWS values for the entire LOS. Diagnoses of HF, MI, and AKI were identified using International Classification of Diseases, Tenth Revision billing codes available in the EHR. 

### Statistical Analysis

Statistical analysis was performed in R v3.6.1 (R Foundation for Statistical Computing) using RStudio environment v1.1.456. The participants were first stratified into continuous VS monitoring and contemporaneous controls as intermittent VS monitoring groups. Sequential random nearest neighbor matching with a propensity score ratio of 1:3 and with a caliper of 0.25 [25,26] was used to balance the potential factors affecting the outcome including selection bias. To create propensity score–matched pairs, we performed random 1-to-3 matching using the MatchIt package in R, in which the control variables in our study were race, gender, ethnicity, age, quarter-year of admission, BMI, Charlson Comorbidity Index (CCI), patient’s primary insurance type, and source of admission [27]. The variable selection for propensity score matching was based on feedback from clinical expertise. To adjust for the effect of time and variability of the continuous wireless monitoring device use, the quarter-year of admission was used as a covariate in the model. Standardized differences were used as indicators of intergroup balance. If the standardized differences were <10%, the covariates between the 2 groups were considered well-balanced.

For summary statistics, categorical variables were described as numbers and percentages, and non-normally distributed continuous variables were described as median and IQRs.

Primary, secondary, and exploratory outcomes were compared between groups and subgroups. Multivariable logistic regression analysis was performed to detect differences in outcomes between groups. Initially, restricted cubic splines were included with age and CCI to relax the linearity assumptions of the multivariable logistic regression model. As the nonlinear components of age and CCI were not statistically significant, the splines were removed from the variables. The multivariable logistic regression adjusted model was controlled for race, gender, ethnicity, age, BMI, patient’s primary insurance type, source of admission, and CCI. The *P* value for significance level within the primary and secondary outcome analyses was set at .004 for each group of analyses after Bonferroni correction for multiple comparisons.  

To test the influence of unobserved confounding, we used an E-value analysis to quantify the potential implications of unmeasured confounders. E-value is defined as the minimum strength of an association on the risk ratio scale that an unmeasured confounder would need to have with both the exposure and the outcome, conditional on the measured covariates, to fully explain away a specific exposure-outcome association. 

We further performed multivariate logistic regression analysis for the composite outcome on the whole study cohort (n=7971) to assess the effect of continuous monitoring sensitivity after controlling for race, gender, ethnicity, age, BMI, and CCI as a sensitivity analysis.

## Results

**Figure 1:**
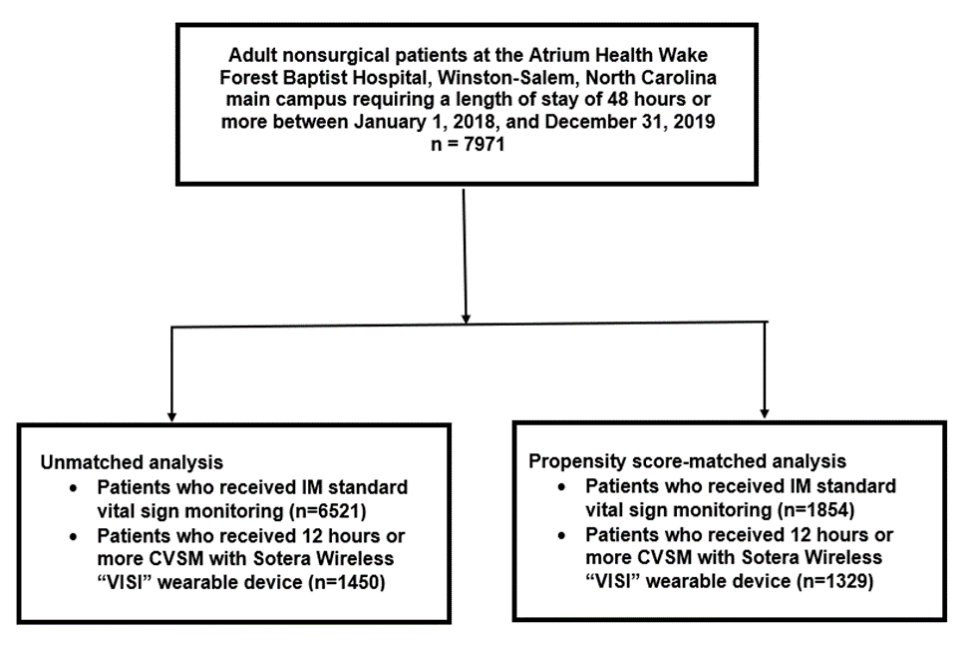
CONSORT (Consolidated Standards Of Reporting Trials) diagram of the retrospective propensity-matched analysis of continuous vital sign monitoring (CVSM) versus intermittent vital sign monitoring (IM) in primary medical patients at an academic tertiary medical center from January 1, 2018, to December 31, 2019.

We identified 7971 patients who met inclusion criteria (Figure 1). Over the study period, 1450 patients received 12 hours or more of continuous VS monitoring, compared with 6521 contemporaneous controls who received intermittent VS monitoring (Table 1).

**Table 1:** Baseline patient demographics and characteristics of the retrospective propensity-matched analysis of continuous versus intermittent vital sign monitoring of primary medical patients at an academic tertiary medical center from January 1, 2018, to December 31, 2019.

Characteristics		Unmatched cohort analysis					Propensity score–matched cohort analysis			
		Continuous vital sign monitoring (n=1450)	Intermittent vital sign monitoring (n=6521)	SMD^a^		Continuous vital sign monitoring (n=1329)		Intermittent vital sign monitoring (n=1854)	SMD	
**Quartile (Q)-year, n (%)**					1.936					0.107
	Q1-2018	411 (28.3)	417 (6.4)			374 (28.1)		417 (22.5)		
	Q2-2018	420 (29.0)	452 (6.9)			372 (28)		452 (24.4)		
	Q3-2018	467 (32.2)	528 (8.1)			431 (32.4)		528 (28.5)		
	Q4-2018	30 (2.1)	926 (14.2)			30 (2.3)		87 (4.7)		
	Q1-2019	10 (0.7)	1324 (20.3)			10 (0.8)		26 (1.4)		
	Q2-2019	10 (0.7)	1211 (18.6)			10 (0.8)		44 (2.4)		
	Q3-2019	49 (3.4)	1082 (16.6)			49 (3.7)		119 (6.4)		
	Q4-2019	53 (3.7)	581 (8.9)			53 (4)		181 (9.8)		
Age (years), median (IQR)		61.00 (48.00-72.00)	62.00 (48.00-73.00)	0.012		61.00 (48.00-72.00)		61.00 (46.00-74.00)	0.018	
**Sex, n (%)**					0.082					0.038
	Male	678 (46.8)	3316 (50.9)			643 (48.4)		932 (50.3)		
	Female	772 (53.2)	3205 (49.1)			686 (51.6)		922 (49.7)		
Ethnicity (Hispanic/Latino), n (%)		77 (5.3)	339 (5.2)	0.005		69 (5.2)		96 (5.2)	0.001	
**Racial identity, n (%)**					0.184					0.067
	White	887 (61.2)	4520 (69.3)			861 (64.8)		1256 (67.7)		
	Black or African American	473 (32.6)	1594 (24.4)			386 (29)		484 (26.1)		
	Other	90 (6.2)	407 (6.2)			82 (6.2)		114 (6.1)		
CCI^b^, median (IQR)		3.00 (1.00-5.00)	3.00 (1.00-5.00)	0.055		3.00 (1.00-5.00)		3.50 (1.00-5.00)	0.079	
Hypertension, n (%)		605 (41.7)	2750 (42.2)	0.009		527 (39.7)		699 (37.7)	0.040	
BMI, median (IQR)		27.77 (23.17-32.88)	27.91 (22.99-32.70)	0.001		27.76 (23.19-32.45)		27.80 (22.81-32.73)	0.020	
**Insurance class, n (%)**					0.052					0.034
	Commercial	235 (16.2)	941 (14.4)			212 (16)		273 (14.7)		
	Government	1078 (74.3)	4921 (75.5)			995 (74.9)		1410 (76.1)		
	Other	137 (9.4)	659 (10.1)			122 (9.2)		171 (9.2)		
**Source of admission, n (%)**					0.039					0.015
	ED^c^	1199 (82.7)	5487 (84.1)			1124 (83.2)		1551 (83.7)		
	Non-ED	251 (17.3)	1034 (15.9)			227 (16.8)		301 (16.3)		
**Hospital service, n (%)**					0.146					0.057
	Hospitalist	822 (56.7)	4011 (61.5)			798 (60)		1119 (60.4)		
	General medicine	463 (31.9)	2028 (31.1)			433 (32.6)		573 (30.9)		
	Family medicine	165 (11.4)	482 (7.4)			98 (7.4)		162 (8.7)		

^a^SMD: standardized mean difference.

^b^CCI: Charlson Comorbidity Index.

^c^ED: emergency department.

For propensity matching, 1329 (1329/1450, 91.7%) patients receiving continuous wireless VS monitoring were matched with 1854 (1854/6521, 28.4%) contemporaneous patients receiving intermittent VS monitoring. When propensity matching was not possible, they were excluded from propensity score–matched analysis. Multimedia Appendix 1 shows the patient demographics and clinical characteristics of patients not matched.

Before propensity matching, 365 (365/6521, 5.6%) of intermittent VS monitoring patients had in-hospital mortality or ICU admission compared with 29 (29/1450, 2%) in the continuous wireless VS monitoring group (Multimedia Appendix 2).

**Figure 2:**
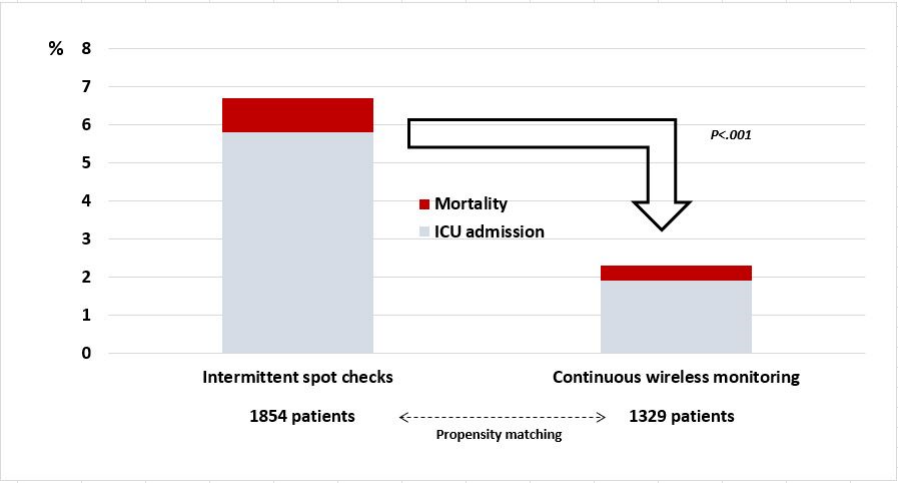
Impact of continuous wireless monitoring on the primary composite outcome of intensive care unit (ICU) admission and mortality from the retrospective propensity-matched analysis of continuous versus intermittent vital sign monitoring in primary medical patients at an academic tertiary medical center from January 1, 2018, to December 31, 2019.

After propensity matching, patients who had intermittent VS monitoring had greater odds of the primary composite outcome, compared with patients who were on continuous VS monitoring (odds ratio 2.79, 95% CI 1.89-4.25; *P*<.001; Table 2, Figure 2).

**Table 2:** Primary and secondary outcomes in the unmatched and matched models from the retrospective propensity-matched analysis of continuous versus intermittent vital sign monitoring in primary medical patients at an academic tertiary medical center from January 1, 2018, to December 31, 2019.

Outcomes	Unmatched model			Propensity-matched model			E value
	Odds ratio (95% CI)	*P* value	Odds ratio (95% CI)		*P* value		
Composite of in-hospital mortality or ICU^a^ admission	2.79 (1.96-4.15)	<.001	2.79 (1.89-4.25)		<.001	2.73	
In-hospital mortality	2.11 (0.99-4.47)	.08	1.91 (0.79-4.31)		.18	2.11	
ICU admission	3.02 (2.06-4.63)	<.001	3.07 (2.03-4.82)		<.001	2.91	
Heart failure	0.96 (0.81-1.14)	.63	1.03 (0.83-1.28)		.77	1.14	
Myocardial infarction	1.22 (0.69-2.38)	.52	1.58 (0.77-3.47)		.23	1.83	
Acute kidney injury	1.01 (0.73-1.03)	.58	0.74 (0.62-1.02)		.06	1.60	
RRT^b^ activation	1.05 (0.84-1.22)	.92	0.94 (0.75-1.19)		.62	1.21	

^a^ICU: intensive care unit.

^b^RRT: rapid response team.

**Figure 3:**
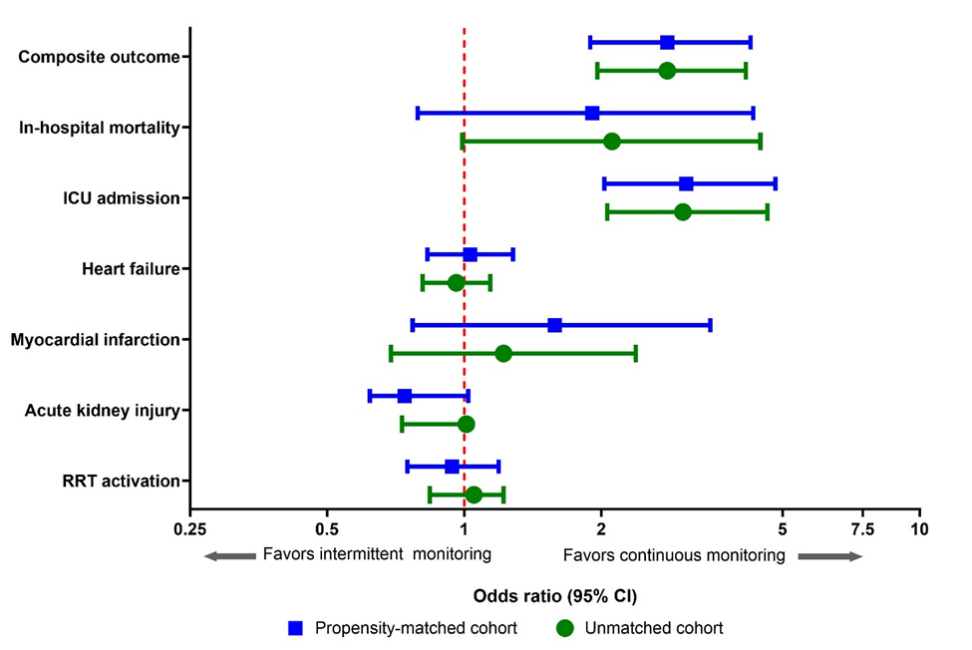
Forest plot of odds ratios (95% CIs) of primary and secondary outcomes for patients receiving intermittent monitoring vs continuous wireless vital sign monitoring from the retrospective propensity-matched analysis in primary medical patients at an academic tertiary medical center from January 1, 2018, to December 31, 2019. ICU: intensive care unit; RRT: rapid response team.

The primary composite outcome in the matched cohort was primarily driven by ICU admission (in-hospital mortality: 5/1329, 0.4% vs 17/1854, 0.9%; ICU admission: 25/1329, 1.9% vs 106/1854, 5.7%). Secondary outcome odds ratios (in-hospital mortality, ICU admission, HF, MI, AKI, and RRT activation) are shown in Table 2 and Figure 3. Intermittently VS-monitored patients had significantly increased odds of in-hospital mortality and ICU admission compared with continuous wireless VS monitoring. Hospital LOS, ICU LOS, and median EWS during the LOS are shown in Table 3.

**Table 3:** Table 3. Exploratory outcomes for propensity-matched cohorts from the retrospective propensity-matched analysis of continuous versus intermittent vital sign monitoring in primary medical patients at an academic tertiary medical center from January 1, 2018, to December 31, 2019.

Outcomes	Continuous vital sign monitoring (n=1329)	Intermittent vital sign monitoring (n=1854)	*P* value
Hospital LOS^a^ (days), median (IQR)	3.12 (2.07-5.25)	3.21 (2.09-5.30)	.80
ICU^b^ LOS (days), median (IQR)	3.32 (2.01-4.75)	2.45 (1.73-4.00)	.10
EWS^c^ during LOS, median (minimum-maximum)	2.00 (1.00-9.00)	1.00 (0.00-9.00)	<.001

^a^LOS: length of stay.

^b^ICU: intensive care unit.

^c^EWS: early warning score.

Multimedia Appendix 3 shows the covariate balance plot before and after propensity score matching between the continuous monitoring cohort and intermittent monitoring cohort. A caliper width of 0.25 was used for the propensity match.

## Discussion

### Principal Findings

In this retrospective propensity-matched analysis, the standard of care intermittent VS monitoring of adult medical ward patients was associated with nearly 3-fold greater odds of ICU admission or in-hospital mortality as compared with continuous VS monitoring. Our work is novel in that this is, to the best of our knowledge, the first propensity-matched study of continuous VS monitoring in the medical ward. Primary medical admissions are inherently different from surgical ones. Medical patients often have multiple acute problems related to single or multiple intersected disease processes.

### Comparison With Prior Work

Our findings corroborate several before-and-after studies mainly conducted with surgical cohorts and are additionally supported by a recently published propensity-matched study by our group in a primary surgical population [28-30]. We hypothesized that, through earlier detection with continuous VS monitoring, patients were able to receive clinical attention sooner, thereby precluding severe clinical deterioration requiring ICU admission. This hypothesis is supported by similar RRT activation rates but greater odds of ICU admission of patients receiving intermittent VS monitoring.

We report a significant difference in favor of continuous monitoring for the primary composite outcome; however, this composite is only from ICU admissions. When compared with a recently published national database study of unplanned ICU admissions of patients who received standard of care intermittent monitoring, we had a greater percentage of patients in our intermittent monitoring arm transfer to the ICU (2.5% vs 5.8%) [31]. Baseline characteristics, including median CCI, were similar; however, the national study included patients with COVID-19 (2021), while our study examined a prepandemic cohort (2018-2019). Moreover, hospitals in the national database varied significantly from ours in terms of size, teaching status, and regional location, which could have influenced outcomes.

### Limitations

Our study was limited primarily by its retrospective approach. First, although propensity matching allowed us to match patients on baseline covariates, we were unable to match based on admission diagnosis or ongoing changes in a patient’s hospital course. We were unable to match on admission diagnosis due to limitations in our EHR data (eg, admission diagnosis missing, inaccurate, or incorrect). Changes over the hospital course occurred after receiving the intervention (continuous versus intermittent monitoring); therefore, the changes were not used to match patients. The inability to match admission diagnosis is a major limitation, but we feel this is offset by our hospital’s triage system by bed availability, which is agnostic to monitoring strategy. The use of continuous wireless VS monitoring or intermittent VS monitoring was not based on patient condition, but it is possible that, over time, patients admitted to continuous VS monitoring units may have represented a subset of clinically different patients from their intermittent VS monitoring counterparts. That is, patients receiving intermittent VS monitoring may have initially been sicker, or more continuous wireless VS monitoring use may have been deployed over a time course to a healthier group. To account for these differences, cohorts were matched on a combination of baseline covariates present on admission along with every quarter-year of admission to adjust for time variance of continuous wireless VS monitoring use throughout our large hospital system. The difference between the number of patients receiving continuous VS monitoring in 2018 compared with 2019 is related to an issue that occurred with the continuous VS monitoring server. Our approach with propensity matching was set at a caliper of 0.25, and the drop-off for continuously monitored patients does demonstrate that patient groups may be different at baseline within the defined caliper. Most of the imbalance was due to the time of year of admission and CCI, which was matched adequately. An increase in the caliper from 0.25 to 0.35 did not show any significant change in the matched cohort number. Second, we relied on reported rates of conditions from discharge summaries and administrative codes, which have their inherent flaws.

Third, our large administrative data set does not have granular information that would allow a deeper understanding of the mechanisms of favorable outcomes with continuous VS monitoring. This includes the number of and reasons for alarms and false alarms as well as the VS captured by the continuous devices. Similarly, we had limited information on EWS values at the time of RRT activation but did include median, minimum, and maximum EWS and absolute numbers of RRT activations for the entire LOS for both groups. A smaller pilot from the same institution and devices showed a rate of 2.3 alarms per patient per day and a significant decrease in RRT calls, from 189 to 158 per 1000 discharges after implementation using a before-and-after study design [21].

### Future Directions

Interesting future outcome research could include whether continuous VS monitoring was associated with changes in medical management, including the timing of antibiotics, discontinuation of supplemental oxygen, and antihypertensive prescriptions. Future work should consider the limitations of this study in order to strengthen the approach, including admission diagnosis matching, granular VS capture, and comparisons between monitoring strategies.

### Conclusion

The ability to continuously monitor patient VS outside of the critical care unit or emergency department using a wearable and wireless device represents a significant technological advancement. However, the quality of evidence for the benefit of continuous VS monitoring is low to moderate, and most studies have been performed in the postoperative setting with before-and-after comparisons. This is the first propensity-matched study using a large data set with contemporaneous controls and a focus on patients with primary medical problems. The inherent differences between a primary surgical population, where nearly all continuous postprocedure monitoring research has been conducted, and a primary medical population is paramount to the significance of this work.

In conclusion, patients receiving intermittent VS monitoring had nearly a factor-of-3 increase in the odds of ICU transfer and in-hospital mortality compared with those receiving continuous VS monitoring. Prospective interventional studies are necessary to confirm the impact of continuous VS monitoring on morbidity and mortality.

## Appendix

Multimedia Appendix 1 Patient demographics and clinical characteristics of the patients who had no match versus propensity-matched patients for each monitoring strategy from the retrospective propensity matched analysis of continuous versus intermittent vital sign monitoring in primary medical patients at an academic tertiary medical center from January 1, 2018, to December 31, 2019.

Multimedia Appendix 2 Absolute values of outcomes measures for the study cohort from the retrospective propensity-matched analysis of continuous versus intermittent vital sign monitoring in primary medical patients at an academic tertiary medical center from January 1, 2018, to December 31, 2019.

Multimedia Appendix 3 Covariate balance plot before and after propensity score matching between the continuous wireless vital sign monitoring cohort and the control monitoring cohort from the retrospective propensity-matched analysis of continuous versus intermittent vital sign monitoring in primary medical patients at an academic tertiary medical center from January 1, 2018, to December 31, 2019. Caliper width of 0.25 was used for the propensity match.

## Abbreviations

AKI: acute kidney injury
BP: blood pressure
CCI: Charlson Comorbidity Index
EHR: electronic health record
EWS: early warning score
HF: heart failure
HR: heart rate
ICU: intensive care unit
IRB: institutional review board
LOS: length of stay
MI: myocardial infarction
RR: respiratory rate
RRT: rapid response team
SpO2: oxygen saturation
VS: vital sign
