# Supplementary material for: Impact on Patient Outcomes of Continuous Vital Sign Monitoring on Medical Wards: Propensity-Matched Analysis
Source: J Med Internet Res. 2025 Mar 11;27:e66347. doi: 10.2196/66347 (PMC11937710; doi:10.2196/66347)
Supplement: Multimedia Appendix 1 [file jmir_v27i1e66347_app1.docx]

|  | **Excluded (no-match) Intermittent vital sign monitoring, (n= 4667)** | **Propensity matched, Intermittent vital sign monitoring, (n= 1854)** |  | ***P*-Value** |
| --- | --- | --- | --- | --- |
| Quarter Year (%) |  |  |  | <0.001 |
| Q1-2018 | 0 ( 0.0) | 417 (22.5) |  |  |
| Q2-2018 | 0 ( 0.0) | 452 (24.4) |  |  |
| Q3-2018 | 0 ( 0.0) | 528 (28.5) |  |  |
| Q4-2018 | 839 (18.0) | 87 (4.7) |  |  |
| Q1-2019 | 1298 (27.8) | 26 (1.4) |  |  |
| Q2-2019 | 1167 (25.0) | 44 (2.4) |  |  |
| Q3-2019 | 963 (20.6) | 119 (6.4) |  |  |
| Q4-2019 | 400 (8.6) | 181 (9.8) |  |  |
| **PATIENT AGE (years) (median [IQR])** | 59.67 (18.53) | 59.33 (19.96) |  | 0.515 |
| **Sex (%)** |  |  |  | 0.572 |
| Male | 2384 (51.1) | 932 (50.3) |  |  |
| Female | 2283 (48.9) | 922 (49.7) |  |  |
| **Ethnicity (%)** | 243 ( 5.2) | 96 (5.2) |  | 0.99 |
| **RACE (%)** |  |  |  | 0.144 |
| White or Caucasian | 3264 (69.9) | 1256 (67.7) |  |  |
| Black or African American | 1110 (23.8) | 484 (26.1) |  |  |
| Other | 293 (6.3) | 114 (6.1) |  |  |
| **CCI (median [IQR])** | 3.00 [1.00, 5.00] | 3.50 [1.00, 5.00] |  | 0.383 |
| **Hypertension (%)** | 2051 (43.9) | 699 (37.7) |  | <0.001 |
| **BMI (median [IQR])** | 27.93 [23.05, 32.67] | 27.80 [22.81, 32.73] |  | 0.788 |
| **Insurance (%)** |  |  |  | 0.321 |
| Commercial Insurance | 668 (14.3) | 273 (14.7) |  |  |
| Governmental Insurance | 3511 (75.2) | 1410 (76.1) |  |  |
| Other Insurance | 488 (10.5) | 171 (9.2) |  |  |
| **Source of Admission** |  |  |  |  |
| ED | 3909 (83.8) | 1578 (85.1) |  | 0.018 |
| Non-ED | 758 (16.2) | 276 (14.9) |  |  |
| **Hospital Service (%)** |  |  |  | 0.031 |
| Hospitalist | 2892 (62.0) | 1119 (60.4) |  |  |
| General Medicine | 1455 (31.2) | 573 (30.9) |  |  |
| Family Medicine | 320 (6.9) | 162 (8.7) |  |  |

CCI, Charlson morbidity index; BMI, body mass index; ED, emergency department
