# Supplementary material for: Impact on Patient Outcomes of Continuous Vital Sign Monitoring on Medical Wards: Propensity-Matched Analysis
Source: J Med Internet Res. 2025 Mar 11;27:e66347. doi: 10.2196/66347 (PMC11937710; doi:10.2196/66347)
Supplement: Multimedia Appendix 2 [file jmir_v27i1e66347_app2.docx]

|  | **Unmatched Cohort** | | **Propensity-matched Cohort** | |
| --- | --- | --- | --- | --- |
| **Outcome** | **Continuous**  **Vital Sign**  **Monitoring**  **(n = 1,450)** | **Intermittent**  **Monitoring**  **(n= 6,521)** | **Continuous**  **Vital Sign**  **Monitoring**  **(n = 1,329)** | **Intermittent**  **Monitoring**  **(n= 1,854)** |
| Composite Outcome (%) | 29(2.0) | 365(5.6) | 25(1.9) | 107(5.8) |
| In-Hospital Mortality (%) | 6 ( 0.4) | 57 ( 0.9) | 5 ( 0.4) | 17 ( 0.9) |
| ICU Admission (%) | 25 ( 1.7) | 339 ( 5.2) | 25 ( 1.9) | 106 ( 5.7) |
| Heart Failure (%) | 142 ( 9.8) | 612 ( 9.4) | 134 ( 9.9) | 172 ( 9.3) |
| Myocardial infarction (%) | 12 ( 0.8) | 66 ( 1.0) | 10 ( 0.7) | 18 ( 1.0) |
| Acute kidney injury (%) | 124 ( 8.6) | 563 ( 8.6) | 108 ( 8.0) | 146 ( 7.9) |
| RRT Activation (%) | 70 ( 4.8) | 259 ( 4.0) | 68 ( 5.0) | 73 ( 3.9) |
| Hospital LOS, days (median [IQR]) | 3.11 [2.06, 5.19] | 3.15 [2.02, 5.36] | 3.12 [2.07, 5.25] | 3.21 [2.09, 5.30] |
| ICU LOS, days (median [IQR]) | 3.32 [2.01, 4.75] | 2.36 [1.41, 3.70] | 3.32 [2.01, 4.75] | 2.45 [1.73, 4.00] |
| EWS median value during LOS (median [minimum, maximum]) | 2.00 [1.00, 9.00] | 1.00 [0.00, 9.00] | 2.00 [1.00, 9.00] | 1.00 [0.00, 9.00] |

EWS, early warning system; ICU, intensive care unit; LOS, length of stay; RRT, rapid response team
