# Supplementary figures and images for: Impact on Patient Outcomes of Continuous Vital Sign Monitoring on Medical Wards: Propensity-Matched Analysis
Source: J Med Internet Res. 2025 Mar 11;27:e66347. doi: 10.2196/66347 (PMC11937710; doi:10.2196/66347)

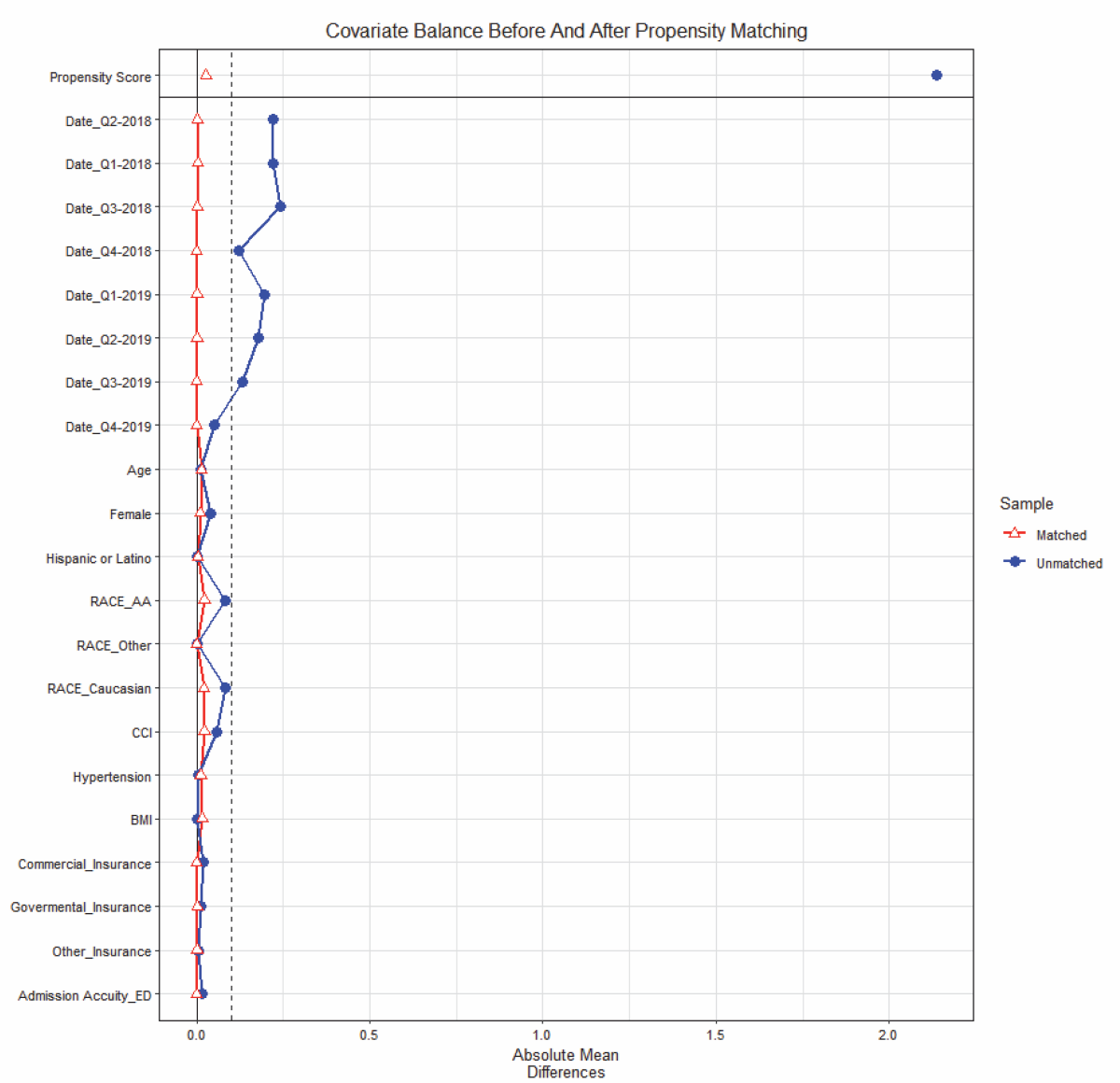

Supplement: Multimedia Appendix 3 [file jmir_v27i1e66347_app3.png]
